# Patients’ Emotional Experiences and Life Changes Following a Diagnosis of Skin Cancer: A Qualitative Study Comparing Melanoma and Squamous Cell Carcinoma

**DOI:** 10.3390/jcm14248891

**Published:** 2025-12-16

**Authors:** Pablo Díaz-Calvillo, Alberto Soto-Moreno, Clara Ureña-Paniego, Juan Ángel Rodríguez-Pozo, Antonio Martínez-López, Salvador Arias-Santiago

**Affiliations:** 1 Dermatology Department, Hospital Universitario Virgen de las Nieves, 18012 Granada, Spainsalvadorarias@ugr.es (S.A.-S.); 2 Ibs.Granada, Instituto de Investigación Biosanitaria de Granada, 18012 Granada, Spain; 3 Skin Cancer Unit, Dermatology Department, Hospital Universitario Virgen de las Nieves, 18012 Granada, Spain; 4 School of Medicine, University of Granada, 18016 Granada, Spain

**Keywords:** melanoma, squamous cell carcinoma, qualitative research

## Abstract

**Background:** Despite advances in diagnosis and treatment, the psychosocial impact of skin cancer remains relatively underexplored. The aim of this study was to explore the emotional experiences of people diagnosed with melanoma and cutaneous squamous cell carcinoma (SCC) and their evolution over time. **Methods**: A qualitative study was conducted using semi-structured interviews. Purposive sampling was employed, taking into account gender, age, tumour type and tumour stage. Interviews were audio-recorded, transcribed verbatim and analysed thematically. **Results:** Thirty-six patients were recruited (18 with melanoma and 18 with SCC). Overall, 61.1% were men and the mean age was 63.8 years (SD 10.8). The analysis revealed three main themes: feelings, relationships and life changes. Patients in both groups experienced a range of emotions following diagnosis, such as anxiety, relief and fear of recurrence. Family dynamics played a crucial role in patients’ experiences, acting as both a source of support and a cause of concern. The diagnosis prompted changes in everyday life, affecting work, daily activities and patients’ sense of identity. **Conclusions:** The diagnosis of melanoma or SCC has a profound emotional and existential impact on patients. Personalised care is crucial to address their evolving concerns and information needs. Further research is needed on the long-term impact of skin cancer and the benefits of psycho-oncological support. Incorporating patients’ perspectives into current skin cancer guidelines should be considered.

## 1. Introduction

Skin cancer is one of the most common malignant neoplasms in humans, and its incidence continues to rise worldwide [1,2]. It is broadly divided into melanoma and non-melanoma skin cancer (NMSC), with NMSC mainly comprising basal cell carcinoma (BCC) and squamous cell carcinoma (SCC). Although melanoma accounts for only around 1% of skin cancers, it causes the majority of skin cancer-related deaths [3]. Advances in early detection and treatment have improved survival, but the global burden of melanoma is expected to increase in the coming decades as a result of ageing populations and cumulative ultraviolet radiation exposure [2].

Surgical excision remains the primary therapeutic approach for melanoma, and in early-stage disease, cure rates are generally high. However, patients with advanced melanoma and lymph node or distant organ involvement require additional interventions such as lymphadenectomy, immunotherapy or targeted therapies [4]. The introduction of immune checkpoint inhibitors and targeted therapies has significantly reduced melanoma mortality and transformed the prognosis of many patients with advanced or metastatic disease [5]. Consequently, an increasing number of individuals live for many years after their initial diagnosis and treatment, transitioning into a long-term survivorship phase.

SCC is the second most common skin cancer after BCC and represents the most clinically relevant keratinocyte carcinoma in terms of potential morbidity and mortality [6]. In Spain, the estimate incidence of SCC is 38.1 cases per 100,000 person-years and with a mortality rate of 1.10 per 100,000 person-years [7]. While many tumours follow an indolent course, SCC can grow rapidly and carries a significant risk of local recurrence and metastasis, particularly in the presence of high-risk features such as immunosuppression, increased tumour thickness or poor differentiation [8]. Surgery is the treatment of choice for most cases and may be complemented by radiotherapy in patients with positive margins or other high-risk factors, whereas systemic immunotherapy is increasingly used in locally advanced or metastatic disease [9]. As with melanoma, improvements in diagnosis and treatment have contributed to a growing population of SCC survivors, many of whom are older adults with multiple comorbidities and repeated interventions on chronically sun-damaged skin.

Beyond oncological outcomes, the diagnosis of skin cancer can profoundly affect patients’ lives and those of their families. High survival rates mean that patients often undergo long-term follow-up, transforming skin cancer into a chronic condition that can affect patients’ lifestyles and social and professional activities. In addition to treatment, regular dermatological and oncological visits, repeated diagnostic tests and procedures, and the possibility of developing new primary tumours or metastases sustain a sense of ongoing vulnerability [10]. Elevated levels of fear of cancer recurrence and disease progression have been associated with depression and anxiety, reduced quality of life and greater healthcare utilisation across different cancer types, including melanoma [11]. However, psychosocial experiences are unlikely to be homogeneous across skin cancer types. Melanoma tends to affect a relatively younger population and is often perceived as a potentially lethal disease even in early stages, whereas SCC more frequently affects older patients who may already be coping with chronic illnesses, functional limitations and caregiving needs [7]. These differences in age, comorbidities, tumour location and treatment intensity may shape how patients interpret their diagnosis, the meaning they attribute to follow-up, and the kinds of support they require.

Qualitative research offers a unique avenue to explore how patients understand and live with skin cancer, complementing quantitative studies focused on symptoms, quality-of-life scores or survival. A qualitative systematic review by Bath-Hextall et al. highlighted that patients with skin cancer frequently report fear of recurrence, uncertainty about the future, body image concerns, impacts on work and social life, and unmet information needs [12]. However, most of the available qualitative literature has either focused on melanoma or has examined skin cancer as a single entity without clearly distinguishing between melanoma and keratinocyte carcinomas [12]. SCC remains comparatively underexplored despite its potential for aggressive behaviour, treatments that may be disfiguring or functionally impairing, and frequent occurrence in older, frail or immunosuppressed patients [6]. Consequently, little is known about whether the emotional trajectories, fears and perceived life changes in patients with SCC differ from those of patients with melanoma, or about the specific psychosocial needs that might arise in each group.

A better understanding of these aspects is essential for designing patient-centred models of care and to integrate psycho-oncological and supportive interventions into routine skin cancer management. Clarifying similarities and differences between melanoma and high-risk SCC may help clinicians tailor information, communication and follow-up strategies according to patients’ expectations, perceived risks and life circumstances rather than solely tumour biology. To address these gaps, this qualitative study investigates the emotional experiences and fears of individuals diagnosed with melanoma or high-risk SCC, examining how these evolve over time and influence relationships and perceived changes in everyday life. By comparing patients with melanoma and high-risk SCC, we seek to generate knowledge that can inform clinical practice guidelines and survivorship programmes for people living with skin cancer.

## 2. Materials and Methods

### 2.1. Study Design

This qualitative study was conducted at the Virgen de las Nieves University Hospital using semi-structured interviews with patients diagnosed with melanoma or SCC. The Consolidated Criteria for Reporting Qualitative Research (COREQ) checklist was used to guide the design and reporting of the study [13].

### 2.2. Participants

Inclusion Criteria:

- Patients with confirmed diagnosis of melanoma for between 1 and 3 years before the inclusion, treated at the Skin Cancer Unit of the Dermatology Department of the Virgen de las Nieves University Hospital.
- Patients with confirmed diagnosis of SCC for between 1 and 3 years before the inclusion, treated at the Skin Cancer Unit of the Dermatology Department of the Virgen de las Nieves University Hospital. SCC cases treated in this unit are classified as high-risk SCC, categorised according to the Brigham and Women’s Hospital staging system [14] as T2a, T2b, T3 or as having disseminated disease.
- Age ≥ 18 years.
- Ability to understand and participate in a verbal conversation.
- Provision of written informed consent to participate in the study.

Exclusion Criteria:

- Physical or mental conditions that did not allow completion of an interview.
- Lack of written informed consent form.

### 2.3. Sampling Method

Purposive sampling was conducted to achieve maximum diversity based on the following segmentation criteria to generate different profiles:

- Gender: male, female.
- Age (years): <65, ≥65.
- Tumour type: melanoma, SCC.
- Tumour stage: early (stage I, stage II), advanced (stage III, stage IV) [15].

To recruit participants, patients attending the Skin Cancer Clinic of the Dermatology Department at Virgen de las Nieves University Hospital were approached after their consultation and briefly informed about the study. They were asked whether they would be willing to participate in an interview about their experiences with skin cancer. Patients who expressed interest received an information sheet and an informed consent form; for those who agreed to participate, an interview was scheduled and conducted. Recruitment was stopped once all predefined sampling groups were represented and interview data no longer generated new relevant information.

### 2.4. Data Collection, Variables and Sources of Information

Data were collected through individual semi-structured interviews conducted between June and December 2024 by two members of the research team (P.D.-C., C.U.-P.) with prior experience in clinical interviewing and training in qualitative methods. A semi-structured interview guide was specifically developed for this study after a review of the literature on the psychosocial impact of skin cancer and discussion within the Skin Cancer Unit. The guide consisted of a small number of broad, open-ended questions inviting participants to describe how they experienced being told that they had skin cancer, their main fears at that time, the people they thought about when they received the result, how their view of the disease had evolved over time and how their life had changed since the diagnosis. Additional short follow-up prompts were used when needed to clarify or deepen issues raised by participants.

The initial version of the guide was discussed by the research team to ensure clinical relevance and clarity. During the first interviews, the guide was reviewed on a weekly basis and minor wording changes and reordering of questions were made to improve flow and comprehension while preserving the core content. The final version of the semi-structured interview guide is provided in Supplementary Materials (Table S1).

The interviews were conducted individually, face-to-face, in a quiet, private consultation room within the Dermatology Department, immediately after the clinical appointment. Only the interviewer and the participant were present, with the door closed to ensure privacy and confidentiality. Interviews lasted 20–45 min. All interviews were conducted in Spanish, audio-recorded and subsequently transcribed verbatim by the interviewer. A second researcher (S.A.-S., A.M.-L.) checked each transcript against the corresponding audio file to ensure accuracy. Finally, the transcripts were anonymised before analysis.

### 2.5. Statistical Analysis

Participants’ socio-demographic and clinical characteristics were summarised using simple descriptive statistics (absolute frequencies and percentages). Given the qualitative design and sample size, no inferential statistical tests were performed.

For the qualitative data, an inductive thematic analysis was conducted following Braun and Clarke’s approach to identify and organise patterns of meaning in the narratives of the interviews [16]. The analysis was conducted with the assistance of NVivo 12.4 (QSR International).

Two researchers (A.S.-M., J.Á.R.-P.) participated in data coding. First, they independently coded a subset of eight interviews. They then met to compare their coding, discuss differences and agree on a common list of codes and definitions, which formed a preliminary codebook. This codebook was discussed with the rest of the research team (A.M.-L., S.A.-S., P.D.-C., C.U.-P.) and refined by consensus. Using the agreed codebook, the same two researchers (A.S.-M., J.Á.R.-P.) coded all remaining interviews. New codes, necessary modifications and any ambiguous segments were discussed in regular research team meetings, and discrepancies were resolved by consensus. Finally, the set of codes and the thematic structure were reviewed and approved by the senior researchers (A.M.-L., S.A.-S.). No formal inter-rater agreement coefficient was calculated; instead, analytical rigour was sought through initial double coding, iterative discussion and consensus-based interpretation.

### 2.6. Ethical Considerations

The study involved data collection from medical records and interviews, without any interventions that posed additional risks to participants. Informed consent was obtained from all participants, in accordance with current regulations on personal data protection and digital rights. Provision of written informed consent was a prerequisite for participation and was verbally reconfirmed at the beginning of each interview.

This study was conducted in accordance with the Declaration of Helsinki and was approved by the Ethics Committee of the Hospital Universitario Virgen de las Nieves (protocol code CUALICP-01).

## 3. Results

Thirty-six participants (18 with melanoma, 18 with SCC) were recruited (Table 1). The analysis identified three main themes: feelings, relationships and life changes.

### 3.1. Feelings

#### 3.1.1. Before Diagnosis (Table 2)

Patients experienced mixed emotions upon noticing a skin lesion. Worry was common when previous knowledge or experience raised suspicion, whereas others felt tranquil, attributing lesions to non-cancerous causes.

**Table 2:** Patient testimonies about their feelings before the diagnosis.

Before Diagnosis	
Worry	“I noticed a lump. I went to my general practitioner because I had had a similar mole before, and they removed it without any issues. I felt it was something similar, but since it was bigger, the general practitioner said he couldn’t remove it and that it would be better for a dermatologist to check it. When the dermatologist wanted to remove it so quickly, I suspected something… this can’t be good.” P1: melanoma, male, <65 years, early-stage
Tranquillity	“I didn’t feel unwell because what I had was a wart on my back that was oozing.” P29: melanoma, female, <65 years, advanced-stage
	“I was cleaning the garden, and I got pricked by a tree branch. From that moment, it started getting worse until I had to come in.” P4: SCC, male, ≥65 years, early-stage

SCC: squamous cell carcinoma.

#### 3.1.2. At Diagnosis (Table 3)

Patients frequently reported shock upon hearing the word “cancer”. Concern about the disease’s severity and treatment followed, although some reacted with indifference or acceptance, depending on prior knowledge or previous experiences with cancer.

**Table 3:** Patient testimonies about their feelings at diagnosis.

Before Diagnosis	
Shock	“I was in shock. I didn’t know how to react. I didn’t expect it at all. I’d had it for a while. You don’t understand, you don’t think it’s something serious…” P13: melanoma, female, <65 years, early-stage
Worry	“Since the doctors had already removed a lesion before, at first I thought “let’s see”, but when the doctor told me they had to remove lymph nodes and all that, I got really scared because I had read a lot about melanoma.” P19: melanoma, female, <65 years, early-stage
	“It affects you because the word “cancer” means something serious, right? So the first time I felt depressed and anxious, but then as time passed, you accept it.” P24: SCC, male, ≥65 years, advanced-stage
Indifference	“At first, I didn’t even know what it was. I knew I had a mole, but I didn’t understand the severity until they explained that it could be serious depending on the extent.” P11: melanoma, male, <65 years, early-stage
Acceptance	“I don’t usually worry about things until they’re right in front of me. Since they had already removed a similar lesion from my cheek, it didn’t really surprise me.” P22: SCC, male, <65 years, early-stage

SCC: squamous cell carcinoma.

#### 3.1.3. After Diagnosis (Table 4)

After diagnosis, concern about recurrence persisted for many participants, while others coped through resignation or optimism. Some SCC patients became more self-conscious about their condition, and others felt confused about needing close monitoring despite feeling completely healthy. Over time, many participants reported a gradual sense of tranquillity as they adapted to treatment and gained clarity about their disease.

**Table 4:** Patient testimonies about their feelings after diagnosis.

Before Diagnosis	
Worry	“I thought it was something that by removing it, it would go away. I didn’t attach much importance to cancer at first, but then more lesions kept coming, and that’s when I took it seriously.” P10: SCC, male, ≥65 years, advanced-stage
	“I didn’t notice the slightest change in my body. It’s impossible that I’m dying on the inside when I feel so normal.” P22: SCC, male, <65 years, early-stage
Seeking information	“You watch TV, look online, and hear about others, and then you start thinking about what you did and didn’t do… things that happen to everyone. “ P7: melanoma, male, <65 years, early-stage
Avoidance	“I prefer not to think about something new appearing.” P13: melanoma, female, <65 years, early-stage
Resignation	“Now, I feel exhausted and less optimistic. After the first few interventions, I came out motivated, but now I’m feeling more down. I’m not scared of the disease, it’s going to be there… when my time comes, I’ll accept it and that’ll be the end of it.” P20: SCC, male, <65 years, advanced-stage
Tranquillity	“Over time, I’ve become more confident with my illness. The metastases were localised, and they were completely removed. When they told me I’d need another operation on the left side, I was scared again, but this time I was more confident because I knew the steps: if I need surgery, I’ll do it, if I need adjuvant therapy, I’ll do it.” P23: melanoma, female, <65 years, advanced-stage

SCC: squamous cell carcinoma.

#### 3.1.4. Future Concerns (Table 5)

Many patients feared recurrence, whereas others expressed acceptance of their fate. Specific fears included death, suffering and loss of autonomy, as well as aesthetic concerns, especially after multiple interventions.

**Table 5:** Patient testimonies about future concerns.

Future Concerns	
Worry	“I keep thinking that I’ll never be fully cured. I’ll always have this sword over my head, and something could appear at any moment.” P15: melanoma, male, ≥65 years, early-stage
	“Every time I go for a check-up, I feel a little nervous. The night before, I couldn’t sleep.” P26: SCC, female, <65 years, early-stage
Acceptance	“I’m aware of my problem. I feel well cared for by both dermatology and oncology. The only thing I can do is handle each problem as it arises.” P6: SCC, male, <65 years, advanced-stage
	“Some people’s world would fall apart with news like this… but can we predict what will happen tomorrow? I’ve been driving trailers for 40 years, and you can’t prevent what might happen tomorrow. You can’t live thinking like that.” P28: SCC, male, <65 years, early-stage
Fear of death	“You think you could die. Everything crosses your mind, and since I’ve never been sick, I thought of the worst. My world collapsed.” P13: melanoma, female, <65 years, early-stage
Fear of being ill	“When the time comes, it will come. My only fear is that I won’t be able to take care of myself and will burden my family.” P24: SCC, male, ≥65 years, advanced-stage
Fear of suffering	“I’m not afraid of dying. But I am afraid of suffering.” P10: SCC, male, <65 years, early-stage
Fear of losing control	“My main fear was that I would lose my mind.” P17: melanoma, female, <65 years, early-stage
Aesthetic concerns	“When I got the diagnosis, my world collapsed. I imagined myself bald, sick. Later, the treatment was more bearable because with nivolumab, I didn’t lose my hair, which made it easier.” P29: melanoma, female, ≥65 years, advanced-stage
	“The surgery went great, but what bothers me is that I was left with a deep scar, though I know it had to be done that way.” P14: SCC, female, <65 years, early-stage
External opinions	“The area to be treated was huge, seeing the stitches, the wounds, the dressings… Physically, I was fine, but people kept asking me, ‘What happened to you?’” P22: SCC, male, <65 years, early-stage

SCC: squamous cell carcinoma.

### 3.2. Relationships (Table 6)

#### 3.2.1. Family

Family was perceived as a crucial source of support but also a cause of concern, as patients worried about causing distress to loved ones, particularly among women with caring responsibilities for dependent relatives. In addition, family served as a source of memories, often bringing to mind previous experiences of cancer in relatives.

**Table 6:** Patient testimonies regarding relationships.

Family	
Support	“One of the greatest sufferings I have is my wife. She’s suffering with me and enduring the disease.” P12: SCC, male, ≥65 years, advanced-stage
Concern	“Well, mainly you think about your family… Besides my wife and kids, I thought about my elderly parents. I imagined they’d have a really hard time if something happened to me.” P3: melanoma, male, <65 years, early-stage
	“When I was diagnosed with cancer, I thought about my kids. I hope nothing happens to me, for their sake.” P26: SCC, female, <65 years, early-stage
Source of memories	“A colleague of mine had something like this on his skin. He caught it, and he died.” P28: SCC, male, <65 years, early-stage
Indifference	“I didn’t think about anyone when I was told the diagnosis because I didn’t think it was anything to worry about.” P2: SCC, male, ≥65 years, early-stage
Healthcare professionals	
Trust	“The first time I went to the dermatologist I was quite reassured. Knowing the treatment plan and my options gave me peace of mind.” P5: melanoma, female, <65 years, early-stage
	“I feel very well taken care of in dermatology and oncology. I know I have this problem, and the only thing I can do is try to resolve it whenever it reappears.” P6: SCC, male, <65 years, advanced-stage
Negative experiences	“I had this little crust there, and after a while, a lump started to form… But like I said, they didn’t directly mention cancer… Later, I figured it out.” P18: SCC, male, <65 years, early-stage
	“I had a small red lump in my tear duct. I went to dermatology, and they did a graft. Three days later, it was gone. I went back, and they said it was nothing. Then, it started getting red again. The doctor said: ‘No, it’s not important.’ Well, okay. A few months later, they had to operate again. This time, I lost my eye.” P20: SCC, male, <65 years, advanced-stage

SCC: squamous cell carcinoma.

#### 3.2.2. Healthcare Professionals

Patients generally expressed trust in healthcare professionals, although some described negative experiences related to communication or treatment delays.

### 3.3. Life Changes (Table 7)

#### 3.3.1. Precautions and Surveillance

Following diagnosis, patients adopted precautionary measures, especially regarding sun exposure, and were vigilant about monitoring new skin lesions.

**Table 7:** Patient testimonies about life changes.

Precautions and surveillance	“You know, it’s minimal or no sun, high protection, and taking care of yourself. I used to be reckless. Now, when I play sports, I wear a shirt and lots of sunscreen.” P1: melanoma, male, <65 years, early-stage
	“Now, as soon as I see the sun, I hide. And whenever I see someone out in the sun, I tell them. You’re much more cautious. You think it won’t happen to you, and look at me.” P5: melanoma, female, <65 years, early-stage
	“Now I go out less. In summer, I don’t go to the beach. I’m scared of the sun, and I’ve distanced myself from socializing a bit.” P22: SCC, male, <65 years, early-stage
Lifestyle adjustments	“I used to have certain habits regarding the sun that I’m trying to change, along with lifestyle and diet habits. It’s hard, but I’m working on it, little by little.” P19: melanoma, female, <65 years, early-stage
	“My life is mostly about seeing doctors. But whenever I can, I like to go for walks in the countryside. That’s about it. At my pace, though. My legs can’t take much anymore.” P24: SCC, male, <65 years, advanced-stage
Work-related changes	“The systemic treatment phase felt long because it lasted a whole year, going to the hospital every 15 days. I was on sick leave because I work as a preschool teacher, but I managed well.” P29: melanoma, female, <65 years, early-stage
	“It changes your life. They retired me at 52, and everything’s different now. I had to find other things to keep myself busy.” P20: SCC, male, <65 years, advanced-stage
Daily activities	“The disease made me retire, and the aftermath of surgery has limited me a lot, like when it comes to hanging up the laundry or sweeping. I’ve had to rearrange my house.” P31: melanoma, female, <65 years, advanced-stage
	“I used to go out to the countryside every day, but I don’t anymore. I don’t go out at all now and live with my daughter.” P32: SCC, female, ≥65 years, early-stage
Health-related changes	“After the diagnosis, you go to your checkups, constantly checking yourself… If something itches, you start thinking it could be something serious.” P17: melanoma, female, <65 years, early-stage
	“I’m not very conscious of not having lymph nodes, but when my arm swells, I get really scared. I think, ‘This must mean something else is appearing…’ But when the swelling goes down, I calm down.” P29: melanoma, female, ≥65 years, advanced-stage
Identity	“Now I appreciate the things I didn’t think much of before.” P11: melanoma, male, <65 years, early-stage
	“Mostly, it changed the way I think about some things. It’s like your perspective shifts a little… It makes me see the good in life.” P13: melanoma, female, <65 years, early-stage

SCC: squamous cell carcinoma.

#### 3.3.2. Working and Daily Life

Many patients adjusted their working life or retired early because of the impact of the diagnosis and its treatment. Treatment side effects influenced their ability to perform daily tasks.

#### 3.3.3. Health and Identity

The diagnosis led to heightened health awareness, with constant self-checking and anxiety about new symptoms. Many patients described a shift in values and priorities as a result of their illness, and some SCC patients reported feeling different about their appearance.

## 4. Discussion

The present study offers valuable insights into the emotional experiences, coping mechanisms and life adjustments of patients diagnosed with melanoma and SCC. By exploring patients’ nuanced reactions to diagnosis and treatment, this study highlights common psychological challenges, including worry, acceptance and the impact on relationships and daily life. These findings contribute to a deeper understanding of patient experiences, which can inform tailored psychosocial support strategies and improve patient-centred care.

The diagnosis of skin cancer has a significant impact on patients’ emotional, psychological and social well-being, irrespective of cancer type [12]. However, the nature of this impact varies depending on patients’ prior concerns. Those with heightened anxiety before diagnosis often report relief afterwards, a shift they attribute to online information-seeking behaviours. While such information can alleviate immediate fears, it may also increase stress due to its generic and non-personalised nature, fostering uncertainty [17]. Conversely, patients who were calm or indifferent before diagnosis often experience heightened stress once the diagnosis is confirmed. This highlights the importance of healthcare providers exploring patients’ emotional experiences and existing level of knowledge to effectively mitigate the psychological impact of the diagnosis.

Patients with a history of cancers or other serious illnesses may perceive skin cancer as less significant, viewing it as another challenge in a life already filled with medical hardships [18]. However, this perspective can shift if there is a family history of cancer, even if unrelated to skin cancer, as it evokes memories of negative experiences with relatives. Family dynamics also significantly shape the patient’s journey. While families are frequently viewed as sources of support, particularly in monitoring and detecting new lesions, they can also be sources of concern. Patients, particularly women who assume caregiving roles, often experience anxiety about their family responsibilities [19,20]. Conversely, patients frequently draw comfort from their families, particularly from seeing their children established and successful, which fosters a sense of acceptance and contentment. Notably, SCC patients, who are generally older, tend to display a higher level of acceptance regarding the potential for death, possibly due to a perception of having resolved life’s major tasks [21].

Consistent with previous research, both melanoma and SCC patients encountered difficulties in understanding information provided by healthcare professionals [22,23]. When their questions remained unanswered following consultations, many patients sought information online [24], which often failed to address their specific concerns, exacerbating their anxiety [25]. To address this, healthcare providers must ensure that patient inquiries are thoroughly answered in a clear and personalised manner, as satisfaction with the information received is strongly correlated with improved well-being [22,26].

Interestingly, SCC is not perceived by patients as being as serious as other cancers. While it is true that the prognosis, especially for NMSC, is generally more favourable compared to other common cancers, such as colorectal or breast cancer [27,28,29], this perception can lead patients to feel uncertain or confused about the need for intensive treatment or follow-up, particularly when they feel physically healthy. This paradox is more pronounced in SCC due to the lack of symptoms such as pain or bleeding, which often makes the disease seem less real to the patient [27]. Furthermore, the perception of SCC as a minor malignancy may be perpetuated by healthcare professionals themselves. Some SCC patients reported that their physicians did not use the word “cancer” during diagnosis, contributing to a delayed recognition of the importance of their condition. This phenomenon may stem from the excellent prognosis associated with these tumours, which differs from the general public’s perception of cancer as synonymous with poor prognosis or even death [30]. This paternalistic approach, while likely intended to alleviate immediate distress, underscores the need for healthcare professionals to provide clear, comprehensive information, even in cases of favourable prognosis [31].

Our findings reveal a spectrum of coping strategies among both melanoma and SCC patients, ranging from proactive lifestyle changes to the maintenance of normalcy, underscoring the highly individualised nature of these strategies [32,33]. Patients exhibited increased vigilance over their skin health, consistent with previous research [34], but this heightened awareness was often accompanied by increased anxiety and stress about potential recurrence. This finding aligns with broader literature emphasising the pervasive fear of recurrence among cancer survivors [35,36]. Additionally, it underscores the importance of having a trusted and well-informed professional to consult when they detect any suspicious lesions [31].

Fear of recurrence transcends specific cancer types [37]. However, the experiences of patients evolve differently over time according to the type of skin cancer. Melanoma patients generally exhibit a heightened awareness of their condition from the outset and grasp its severity. As time progresses and they witness their disease remaining dormant with no new lesions emerging, initial apprehensions gradually give way to a sense of calm, though residual fears of recurrence persist. Conversely, SCC patients may experience greater anxiety over time and subsequent follow-up visits due to the chronic nature of their condition. This divergence could be attributed to the distinct biological behaviours of the two cancers. Melanoma typically presents as a solitary lesion, with fewer new lesions detected during follow-up, while SCC is often associated with the chronic development of actinic keratoses and other lesions, keeping patients in a state of ongoing vigilance [38].

The concept of biographical disruption is well-documented in the cancer and chronic disease literature, with patients often experiencing changes in their work, daily activities and appearance, which threaten their personal identity [39]. Many participants in our study reported significant professional changes, such as early retirement or workplace adaptations, as a result of their diagnosis. These changes not only pose economic challenges but also necessitate emotional and social adjustments, as patients navigate shifts in their professional identity. Our findings align with studies that highlight the need for workplace adaptations and psychosocial support for cancer survivors [18,39,40]. Physical limitations, especially among older SCC patients, further underscore the need for comprehensive support systems that address both practical and emotional needs. Changes in appearance, particularly in SCC patients, were more pronounced, likely because SCC often affects highly visible areas such as the face [41]. These findings underscore the multifaceted impact of skin cancer on patients’ functional independence and emphasise the need for holistic models of care [42].

For many patients, the diagnosis of melanoma or SCC can trigger an existential re-evaluation, leading them to reassess their values, priorities and life goals. This process highlights the resilience many individuals exhibit when confronting cancer and underscores the transformative potential of adversity [43].

Our study offers several strengths that add meaningfully to the existing literature. The use of qualitative methods allows for an in-depth exploration of patients’ experiences, providing valuable insights into the psychosocial impact of skin cancer. By including patients with both melanoma and SCC, we offer a comprehensive understanding of the challenges faced across these different cancer types, enhancing the generalisability of our findings. Additionally, by examining various facets of patients’ post-diagnosis lives, we present a holistic perspective, which can inform the development of tailored support interventions and survivorship care plans.

In clinical practice, our results can be translated into concrete actions such as incorporating brief psychosocial assessments into dermatology and skin cancer clinics, explicitly addressing fear of recurrence, clarifying the meaning of “cancer” and prognosis (particularly in SCC), and involving families in education and surveillance when appropriate. Our findings also support the integration of psycho-oncology services into skin cancer care pathways and the co-design, together with patients and caregivers, of targeted interventions (for example, psycho-educational programmes, group or individual psychological support, and digital resources) tailored to high-risk skin cancer survivors. Future research should evaluate the effectiveness and cost-effectiveness of such interventions in different healthcare settings, examine long-term trajectories of emotional adjustment and fear of recurrence in distinct skin cancer subgroups, and identify which patients are most likely to benefit from additional support, ideally through longitudinal and mixed-methods designs.

However, our study has several limitations. The homogeneity of our sample, drawn from specific geographic regions and specialised clinics, may introduce sampling bias, limiting the generalisability of our findings. Future research should aim to include more diverse populations to capture a wider range of experiences. Additionally, the use of self-reported data may introduce recall or social desirability bias, and the cross-sectional nature of the study may not fully capture the long-term impact of a skin cancer diagnosis. Finally, the subjective interpretation inherent in thematic analysis, despite efforts to ensure methodological rigour, remains a potential source of bias.

## 5. Conclusions

Our findings highlight the importance of healthcare professionals’ understanding of patients’ concerns, prior knowledge and information needs in order to provide appropriate support and care. The emotional impact on patients’ lives appears to be largely independent of skin cancer type and needs to be acknowledged and addressed in all patients. However, the needs and preferences over time may differ for melanoma and SCC, as each group experiences their disease, follow-up and risk of new lesions in distinct ways. These insights into patients’ perspectives should be reflected in clinical practice guidelines and survivorship care pathways for melanoma and SCC, including recommendations on information provision, psychosocial assessment and access to psycho-oncological support.

## Figures and Tables

**Table 1:** Participant characteristics.

	Melanoma	Squamous Cell Carcinoma	Total
Age (years)			
<65	12	7	19
≥65	6	11	17
Sex			
Male	9	13	22
Female	9	5	14
Tumour stage			
Early-stage	10	12	23
Advanced-stage	8	6	13
Total	18	18	36

## Data Availability

The data presented in this study are available on request from the corresponding author due to privacy reasons.

## Supplementary Materials

The following supporting information can be downloaded at https://www.mdpi.com/article/10.3390/jcm14248891/s1: Table S1: Semi-structured interview guide.
