# Supplementary material for: Patients’ Emotional Experiences and Life Changes Following a Diagnosis of Skin Cancer: A Qualitative Study Comparing Melanoma and Squamous Cell Carcinoma
Source: J Clin Med. 2025 Dec 16;14(24):8891. doi: 10.3390/jcm14248891 (PMC12733849; doi:10.3390/jcm14248891)
Supplement: Supplementary file 1 [file jcm-14-08891-s001.zip › jcm-4020924-supplementary.pdf]

Table S1. Semi-structured interview guide

	Core open-ended question
Q1	To start, can you tell me about the moment when you were told that you had skin cancer? How did you feel in the days or weeks after that moment?
Q2	What was your main fear when they told you that you had skin cancer?
Q3	Did you think about any particular person when you received the result? Who?
Q4	How has your way of thinking about the disease changed over time and during follow-up?
Q5	How has your life changed since you were diagnosed with melanoma or squamous cell carcinoma? How has this affected your day-to-day life? When you think about the future, what worries you most, and what gives you some peace of mind?
Q6	To finish, is there anything else about your experience with skin cancer that you consider important and that we have not talked about?

In addition to these core questions, the interviewer used short follow-up prompts when needed (e.g., “Could you tell me a bit more about that?”, “How did that affect you?”, “What helped you to cope?”) to clarify or deepen aspects mentioned by the participant.

The original guide was developed and used in Spanish; an English translation is provided below for publication purposes.
